# Dielectric, Piezoelectric, and Vibration Properties of the LiF-Doped (Ba_0.95_Ca_0.05_)(Ti_0.93_Sn_0.07_)O_3_ Lead-Free Piezoceramic Sheets

**DOI:** 10.3390/ma11020182

**Published:** 2018-01-24

**Authors:** Chien-Min Cheng, Kai-Huang Chen, Da-Huei Lee, Fuh-Cheng Jong, Mei-Li Chen, Jhih-Kai Chang

**Affiliations:** 1 Department of Electronic Engineering, Southern Taiwan University of Science and Technology, Tainan 71005, Taiwan; ccmin523@gmail.com (C.-M.C.); dhlee@stust.edu.tw (D.-H.L); fcjong@stust.edu.tw (F.-C.J.); ma230209@stust.edu.tw (J.-K.C.); 2 Department of Digital Game and Animation Design, Tung-Fang Design University, Kaohsiung 82941, Taiwan; d9131802@gmail.com; 3 Department of Electro-Optical Engineering, Southern Taiwan University of Science and Technology, No. 1, Nan-Tai Street, Yungkang District, Tainan 710, Taiwan

**Keywords:** lead-free, piezoceramic sheet, sintering temperature, vibration

## Abstract

By the conventional solid state reaction method, a small amount of lithium fluoride (LiF) was used as the sintering promoter to improve the sintering and piezoelectric characteristics of (Ba_0.95_Ca_0.05_)(Ti_0.93_Sn_0.07_)O_3_ (BCTS) lead-free piezoceramic sheets. Using X-ray diffraction (XRD) and a scanning electron microscope (SEM), the inferences of the crystalline and surface microstructures were obtained and analyzed. Then, the impedance analyzer and d_33_-meter were used to measure the dielectric and piezoelectric characteristics. In this study, the optimum sintering temperature of the BCTS sheets decreased from 1450 °C to 1390 °C due to LiF doping. For the 0.07 wt % LiF-doped BCTS sheets sintered at 1390 °C, the piezoelectric constant (d_33_) is 413 pC/N, the electric–mechanical coupling coefficient (k_p_) is 47.5%, the dielectric loss (tan δ) is 3.9%, and the dielectric constant (ε_r_) is 8100, which are all close to or even better than that of the pure undoped BCTS ceramics. The Curie temperature also improved, from 85 °C for pure BCTS to 140 °C for BCTS–0.07 LiF sheets. Furthermore, by using the vibration system and fixing 1.5 g tip mass at the end of the sheets, as the vibration frequency is 20 Hz, the proposed piezoelectric ceramic sheets also reveal a good energy harvesting performance at the maximum output peak voltage of 4.6 V, which is large enough and can be applied in modern low-power electronic products.

## 1. Introduction

In recent years, lead-free piezoelectric ceramics have attracted considerable attention because of their good piezoelectric properties, high Curie temperature, and low environmental issues. Examples include BaTiO_3_-based materials, (Bi_1/2_Na_1/2_)TiO_3_-based materials, Bi-layered structure materials, tungsten–bronze structures, and (KNaNb)O_3_-based (KNN-based) materials [1,2,3,4,5,6,7,8,9]. Ren et al. [10] have recently reported that a high piezoelectric constant (d_33_) superior to PbZrTiO_3_-based (PZT-based) materials is well established for the ion-modified BaTiO_3_ ceramics by constructing a tri-critical point at room temperature. Therefore, it is worth further exploring the relationship between the phase structure and electric properties of BaTiO_3_-based ceramics for practical applications. Some attempts have been conducted to improve the d_33_ value of lead-free piezoelectric ceramics, and among those methods, a composition-induced phase transition is a very promising method for achieving a higher piezoelectricity for lead-free piezoelectric ceramics [11,12,13].

BaTiO_3_ ceramic reveals a good d_33_ value (about 190 pC/N), but its Curie temperature is too low (about 120 °C), and this will become a serious limitation for general applications [14,15]. Hence, for improving the dielectric and piezoelectric properties of BaTiO_3_ ceramics, many important reports were presented [16,17,18,19,20,21]. The high d_33_ values (350–460 pC/N) of BaTiO_3_ ceramics have been demonstrated by different sintering techniques [22]; these include microwave sintering, ordinary sintering, and two-step sintering. Recently, a surprisingly high d_33_ value of near 620 pC/N has been reported for the BaTiO_3_-based ceramics prepared by inducing a tri-critical point [10], indicating that it is feasible for the BaTiO_3_-based ceramics to replace the lead-based piezoelectric materials.

For the aforementioned considerations, there are several reports concerning the development of low-temperature sintering technologies for BaTiO_3_-based ceramics. Zhu et al. [24] and other authors [25,26,27] have recently reported that the (Ba,Ca)(Ti,Sn)O_3_ ceramics with high d_33_ values are promising candidates for lead-free piezoelectric ceramics. However, it is not easy to find balance between lowering the sintering temperature and maintaining good piezoelectricity. The addition of sintering aids, the appearances of second phases, or variations of phase structure could easily be induced, which lead to an apparent deterioration of piezoelectric properties. An ideal sintering promoter should effectively lower the sintering temperature while maintaining the piezoelectric properties as best as possible. In [26,27], Zhou and Zhao et al. used CuO and Li_2_CO_3_ as the sintering promoters to improve the piezoelectric and dielectric characteristics. The optimum d_33_ values was improved from about 140 pC/N (pure BCTS) to an ultrahigh value of 683 pC/N for (Ba_0.95_Ca_0.05_)(Ti_0.93_Sn_0.07_)O_3_ (BCTS)–CuO_X_ and 485 pC/N for BCTS-Li_2_CO_3_. The optimum k_p_ values was improved from about 11% (pure BCTS) to 55% for BCTS–CuO_X,_ and 39% for BCTS–Li_2_CO_3_. Lithium fluoride (LiF) has a low melting point of 848 °C, and is commonly used in molten salts synthesis. The low melting point of LiF indicates that it may enter a liquid phase during the sintering process, which could probably promote the densification behavior of grains growth at lower temperatures [28,29,30,31]. In this study, a small amount of LiF was added into (Ba_0.95_Ca_0.05_)(Ti_0.93_Sn_0.07_)O_3_ (abbreviated as BCTS–yLiF) piezoceramic sheets for specific purposes; namely, the improvement of sintering ability and piezoelectric characteristics. Using the vibration system, the influence of LiF-doping, tip mass, and vibration frequency on the output peak voltage properties of the BCTS–yLiF sheets were also systematically investigated.

## 2. Experimental Procedures

According to the composition of (Ba_0.95_Ca_0.05_)(Ti_0.93_Sn_0.07_)O_3_ and the conventional solid-state reaction method, a proportional high purity (>99.5%) of raw materials (BaCO_3_, CaCO_3_, TiO_2_, and SnO_2_) were used. After mixing, grinding, and 1300 °C/4 h calcination, commercially LiF (>99.5%) sintering promoters were then introduced into the BCTS calcined powder with the formula of BCTS + y wt % LiF (y = 0, 0.01, 0.04, 0.07 and 0.1 wt %). Then, they were mixed and ball milled for 10 h in ethanol, and dried again. After adding 5 wt % PVA as the binder, the resulting powders were ground again, dried, pressed into sheets with dimensions of 30 × 20 × 0.3 mm^3^ and a pressure of 150 MPa, debindered, and finally sintered at 1300–1510 °C for 2 h. The crystal structure of the sintered sheets was analyzed by X-ray diffraction (XRD) using CuKα radiation (2θ = 20–60°), and the microstructures of the sheets were observed by scanning electron microscopy (SEM). After electrodes painted and polarized (4 kV/mm) in 60 °C silicone oil for 40 min, the piezoelectric constant (d_33_) was measured by a d_33_ meter. Using the impedance analyzer (HP4294A), the relative dielectric constants (ε_r_) and dielectric loss (tan δ) were measured, and electromechanical coupling factors (k_p_) were determined by the resonance and anti-resonance methods, according to the IEEE standards. Finally, the maximum output peak voltages of the piezoelectric sheets were measured by the vibration system.

## 3. Results and Discussion

**Figure 1:**
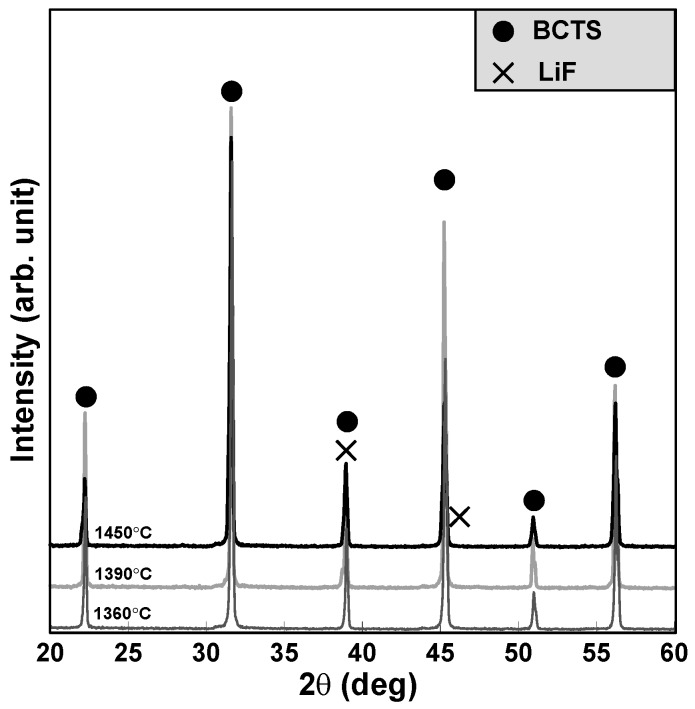
X-ray diffraction patterns of (Ba_0.95_Ca_0.05_)(Ti_0.93_Sn_0.07_)O_3_ (BCTS)–0.07LiF sheets.

**Figure 2:**
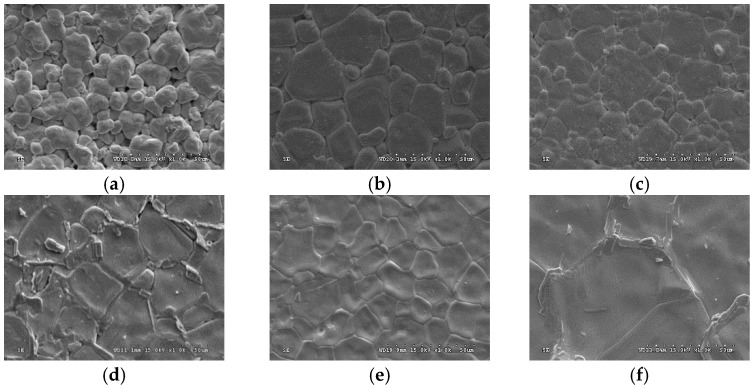

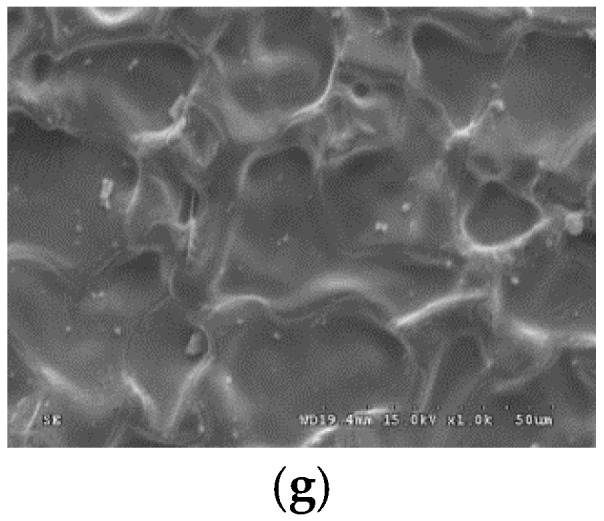
The SEM images of BCTS–yLiF sheets (y = 0 and y = 0.07). (**a**) 1420 °C, y = 0; (**b**) 1450 °C, y = 0; (**c**) 1480 °C, y = 0; (**d**) 1360 °C, y = 0.07; (**e**) 1390 °C, y = 0.07; (**f**) 1420 °C, y = 0.07; (**g**) 1450 °C, y = 0.07.

Figure 1 shows the XRD patterns (2θ = 20–60°) of the (Ba_0.95_Ca_0.05_)(Ti_0.93_Sn_0.07_)O_3_—0.07 wt % LiF ceramics sintered at 1360, 1390 and 1450 °C for 2 h. It can be seen that all of them reveal a pure perovskite structure, and owing to its small amounts and added after calcined process, the peaks of LiF are not obvious and overlaps near 2θ = 40°. The SEM images of the pure BCTS (y = 0) sheets (sintered at 1420, 1450 and 1480 °C for 2 h) are shown in Figure 2a–c, and the SEM images of the BCTS–0.07LiF sheets (sintered at 1360, 1390, 1420 and 1450 °C for 2 h) are also shown in Figure 2d-g, respectively. After comparing these seven figures, we observed that, pure BCTS sheets revealed dense and better grains growth at about 1450 °C. However, for the BCTS–0.07LiF sheets, 1450 °C is already too high for sintering, and a melted surface can be observed (as Figure 2g shown). This means that owing to the addition of 0.07 wt % LiF, the optimum sintering temperature of the BCTS sheets has been lowered from 1450 °C to only 1390 °C. Furthermore, comparing these two sheets with same sintering temperatures of 1420 °C (Figure 2a,f), it can be observed that 1420 °C is too low for pure BCTS (much more pores and small grains), but that for BCTS–0.07LiF, it is too high (very large grains). Thus, LiF will enter a liquid phase (melting point = 848 °C) at the sintering temperature, and it readily plays the role of the sintering promoter.

**Figure 3:**
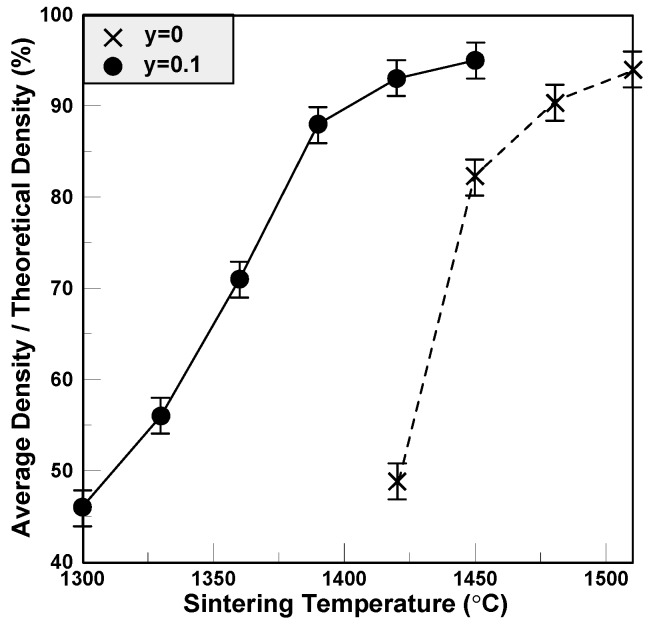
The average density of pure BCTS and BCTS–0.1LiF sheets.

For BCTS ceramics, the lattice constant and theoretical density measured from the XRD patterns shown in Figure 1 are 4.003 Å and 6.04 g/cm^3^, respectively, and the percentages of the measured average densities are shown in Figure 3. It can be seen that for pure BCTS ceramic sheets (y = 0), as the sintering temperature increases, the average density increases, saturates gradually, and equals 93% of the theoretical density at about 1510 °C. However, for the BCTS–0.1LiF sheets, as the sintering temperature increases, the average density increases, and saturates gradually and equals 94% of the theoretical density at only about 1390 °C. This phenomenon means that the LiF was already a good sintering promoter for the BCTS ceramics, which can dense the sheets as well as low down its sintering temperature. However, for obtaining better flexibility of the sheets, more polyvinyl alcohol (PVA) binder were used, and hence, the average density of the sheets was usually smaller than that of the normal specimen.

**Figure 4:**
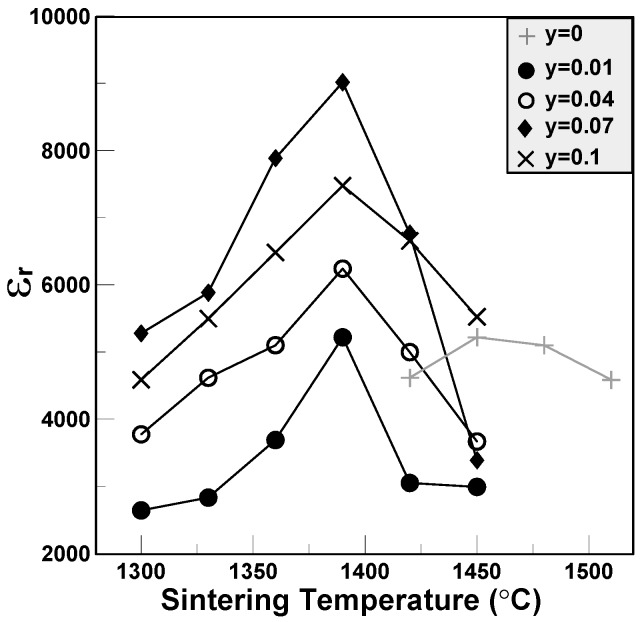
The dielectric constants of the BCTS–yLiF sheets.

The relative dielectric constants of the BCTS–yLiF sheets are shown in Figure 4. It can be seen that for all of the BCTS–yLiF sheets, as the sintering temperature increases gradually, all of the dielectric constants first increase and reach a maximum value, and then decrease gradually. Additionally, pure BCTS reveals the lowest dielectric constants. This means that a too high sintering temperature will cause the dielectric constant to decrease. However, all of the the maximum dielectric constants happened at 1420 °C except for pure BCTS sheets (1450 °C) and BCTS–0.01LiF specimens (1390 °C). The maximum dielectric constants are about 5000 for pure BCTS, and 9000 for BCTS-0.1LiF. This means that even though the additions of only 0.07 wt % LiF, the BCTS sheets will reveal obvious effects, which include: (1) increasing the dielectric constant; and (2) lowering the sintering temperature from 1450 °C to 1390 °C.

**Figure 5:**
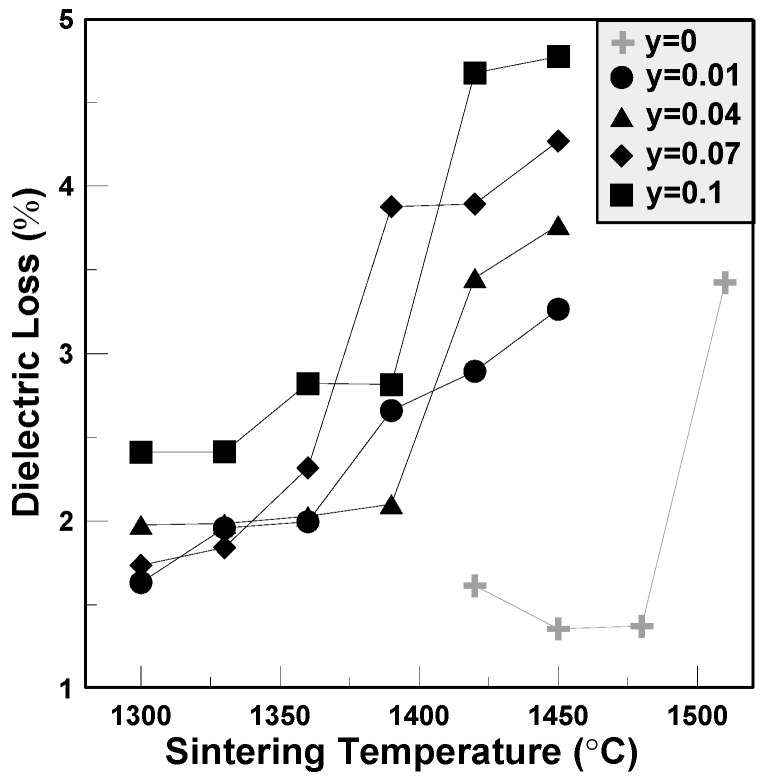
The dielectric loss of the BCTS–yLiF sheets.

The dielectric loss variations of the BCTS–yLiF sheets are shown in Figure 5. For pure BCTS, as the sintering temperature increases, it first decreases to a minimum value, and then increases to about 3.4% for 1510 °C. The optimum dielectric loss is about 1.3%, which happened at 1450 °C. However, for all of the other BCTS–yLiF sheets, the dielectric loss increases as the sintering temperature increase. Compared with pure BCTS sheets, the BCTS–yLiF sheets reveal larger dielectric constants; however, this is accompanied by a higher dielectric loss. This means that too many LiF dopants are not suitable, and will cause an increase of dielectric loss.

**Figure 6:**
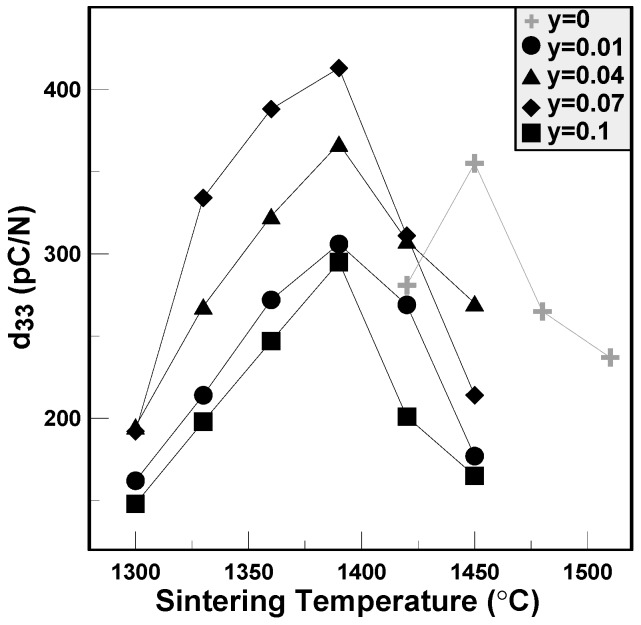
The piezoelectric constants of the BCTS–yLiF sheets.

**Figure 7:**
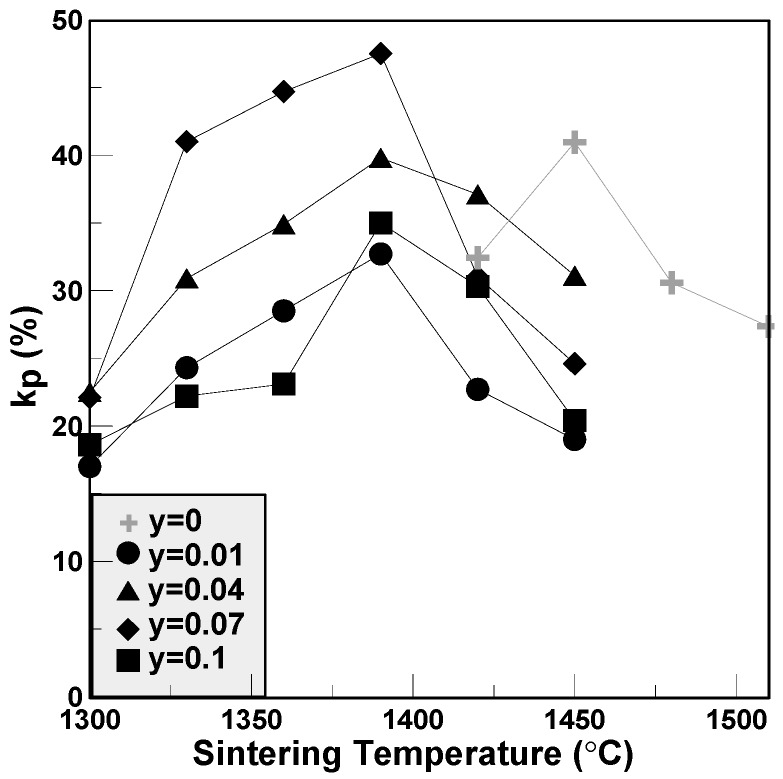
The electromechanical coupling factors of the BCTS–yLiF sheets.

The variations of piezoelectric constants and electromechanical coupling factors of the BCTS–yLiF sheets are plotted in Figure 6 and Figure 7, respectively. It is clear that for all of the specimens, the d_33_ values first increase and reach a maximum value, and then decrease gradually. The maximum d_33_ value of BCTS–0.07LiF is 413 pC/N, and its sintering temperature is only 1390 °C. However, for pure BCTS, the maximum d_33_ value is only 355 pC/N, and sintered at 1450 °C. Hence, it obviously can be found that, owing to the addition of the LiF liquid-phase sintering promoters, the optimal sintering temperature of the BCTS sheets can be reduced significantly about 60 °C, but also piezoelectric characteristics improved. Furthermore, for the electromechanical coupling factors of the BCTS–yLiF sheets, as the sintering temperature increased from 1300 °C to 1450 °C, all of the the k_p_ values revealed the similar trend of d_33_ values. Except for the pure BCTS specimens (which happened at 1450 °C), all of the maximum k_p_ values happened at 1390 °C. For the pure BCTS specimens, the maximum k_p_ value is 0.40, and sintered at 1450 °C; however, for the BCTS–0.07LiF specimens, the maximum k_p_ value is 0.48, and sintered at only 1390 °C.

**Figure 8:**
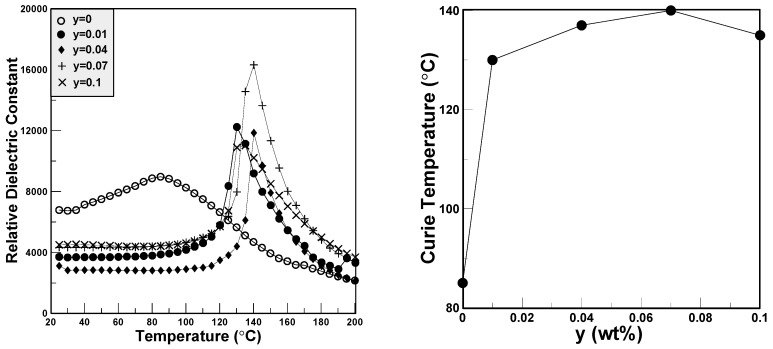
The Curie temperature of the BCTS–yLiF sheets.

The relative dielectric constants measured from room temperature to 200 °C are plotted in Figure 8. It can be seen that the Curie temperature of pure BCTS (y = 0) is about only 85°C, and it increases as the content of LiF increases. The highest Curie temperature happened at y = 0.07, and equaled 140 °C. This means that the addition of LiF has an obvious effect of increasing the Curie temperature by about 55 °C. However, owing to LiF additions, the relative dielectric constants of BCTS–yLiF for room temperature are smaller than those of the pure BCTS sheet.

**Figure 9:**
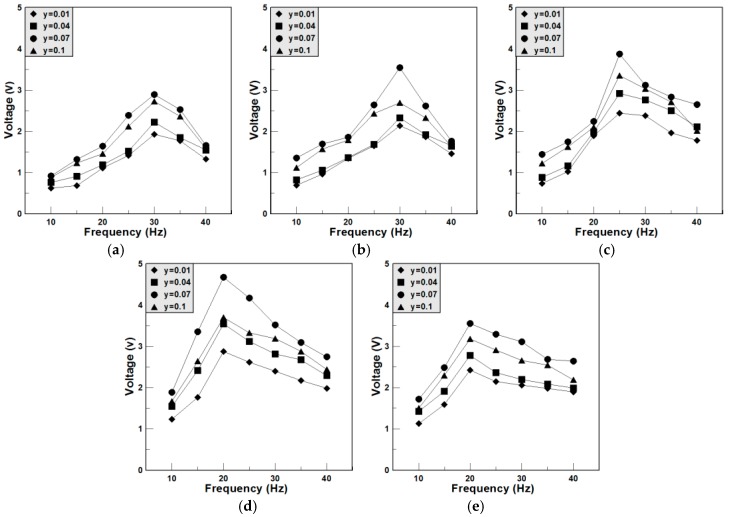
The peak voltage of the BCTS–yLiF sheets for different vibration frequencies. (**a**) Tip mass = 0 g; (**b**) Tip mass = 0.5 g; (**c**) Tip mass = 1.0 g; (**d**) Tip mass = 1.5 g; (**e**) Tip mass = 2.0 g.

By the vibration system, the output voltages of the BCTS–yLiF sheets vibrated at different frequencies and the tip masses were measured and plotted in Figure 9. Additionally, the tip masses were added at the end of the sheets to enhance the effect of vibration. It is noteworthy that, in general, the influencing factors of the best output peak voltage is decided by not only the harmonic frequency and tip mass, but also the flexibility and dimension of the vibration sheets. Hence, it is not certain that the sheets reveal better d_33_ and k_p_ values shown in Figure 6 and Figure 7 will surely obtain better output peak voltages. For different tip masses (0, 0.5, 1, 1.5 and 2 g), all of the maximum output peak voltages happened at BCTS–0.07 LiF sheets, which also corresponds to the best d_33_ and k_p_ values, as shown in Figure 6 and Figure 7. It can be seen from Figure 9a to Figure 9e that the harmonic frequency decreases as the tip mass increases. For the BCTS–yLiF sheets, the best vibration parameters and output results are: y = 0.07; tip mass = 1.5 g; vibration frequency = 20 Hz; and maximum output peak voltage = 4.6 V.

## 4. Conclusions

The influence of LiF additive on the dielectric and piezoelectric properties of BCTS sheets were investigated. The optimal sintering temperature of BCTS sheets was improved from 1450 °C (0 wt % LiF) to 1390 °C (0.07 wt % LiF), and hence the doping of 0.07 wt % LiF can lower the sintering temperature of pure BCTS sheets by about 60 °C. Compared with pure BCTS sheets (sintered at 1450 °C, d_33_ = 355 pC/N, and k_p_ = 0.40), the optimal d_33_ and k_p_ values of the LiF-doped BCTS sheets were up to 413 pC/N and k_p_ = 0.48 for a y = 0.07 specimen. The optimum dielectric loss is about 1.3%, which happened at 1450 °C. However, for other BCTS–yLiF sheets, all of the dielectric losses increased as the sintering temperature increased. The Curie temperature also improved from 85 °C of pure BCTS to 140 °C of the BCTS–0.07LiF sheets. However, with use of a vibration system, when the tip mass is 1.5 g and vibration frequency is 20 Hz, the proposed BCTS–0.07LiF sheets also revealed good energy harvesting performance. The maximum output peak voltage was 4.6 V, which is large enough to be applied in modern low-power electronic products, and act in concert with backend circuits.
